# Arthroscopic Suture Fixation of Os Acetabuli With Absorbable Suture Anchors—A Double-Pulley Technique

**DOI:** 10.1016/j.eats.2022.03.041

**Published:** 2022-07-25

**Authors:** Qing-Feng Yin, Wen-Guang Liu, Ying-Qiang Fu

**Affiliations:** a Department of Orthopedics, The Second Hospital of Shandong University, Jinan, China; b Cheeloo College of Medicine, Shandong University, Jinan, China

## Abstract

Os acetabuli is a bone fragment with unknown origin and isolated at the acetabular rim that may be associated with cam-type femoroacetabular impingement. If this bone fragment is too large and threatens the stability of the hip joint after resection, fixation would be recommended. However, conventional rigid fixation with metal screws has some disadvantages. We describe an arthroscopic suture fixation of the Os acetabulum with absorbable anchors penetrating the bone fragment and secured by tying knots in a double-pulley fashion simultaneously. This technique provides a new feasible solution for the fixation of Os acetabuli, avoiding any metal implants and potential damage to the joint.

Os acetabuli is a bony fragment isolated at the edge of the acetabulum. The cause of os acetabuli is unclear. It has been suggested that it is a residual secondary ossification center, whereas others believe it is a form of nonspecific osteochondritis.^1^^,^^2^ It is currently thought that in young adults, os acetabuli may be a stress fracture caused by repeated overloading on the acetabular rim in patients with femoroacetabular impingement.^3^^,^^4^ The presence of the os acetabuli may be an important factor in aggravating hip impingement and in the development of pain.

The surgical management of os acetabuli has been a matter of debate. The importance of preoperative radiologic evaluation, especially the lateral center-edge angle (LCEA), should be highlighted when considering treatment of os acetabuli. The removal of the small bone fragments that do not interfere with acetabular coverage and hip stability can be performed surgically.^5^ In some cases, the bone fragment is large and may lead to instability after resection, and fixation of the os acetabuli with hollow screws or in combination with sutures is recommended.^4^^,^^6^^,^^7^ In the event of failure of the internal fixation, such as loosening or breakage of the metal implant, the consequences could be catastrophic. In this report, we presented an all-arthroscopic technique to fix os acetabuli with absorbable anchors penetrating the bone fragment and sutures knotted in a double-pulley fashion.

## Surgical Technique (With Video Illustration)

### Preoperative Evaluation and Surgical Plan

**Fig 1:**
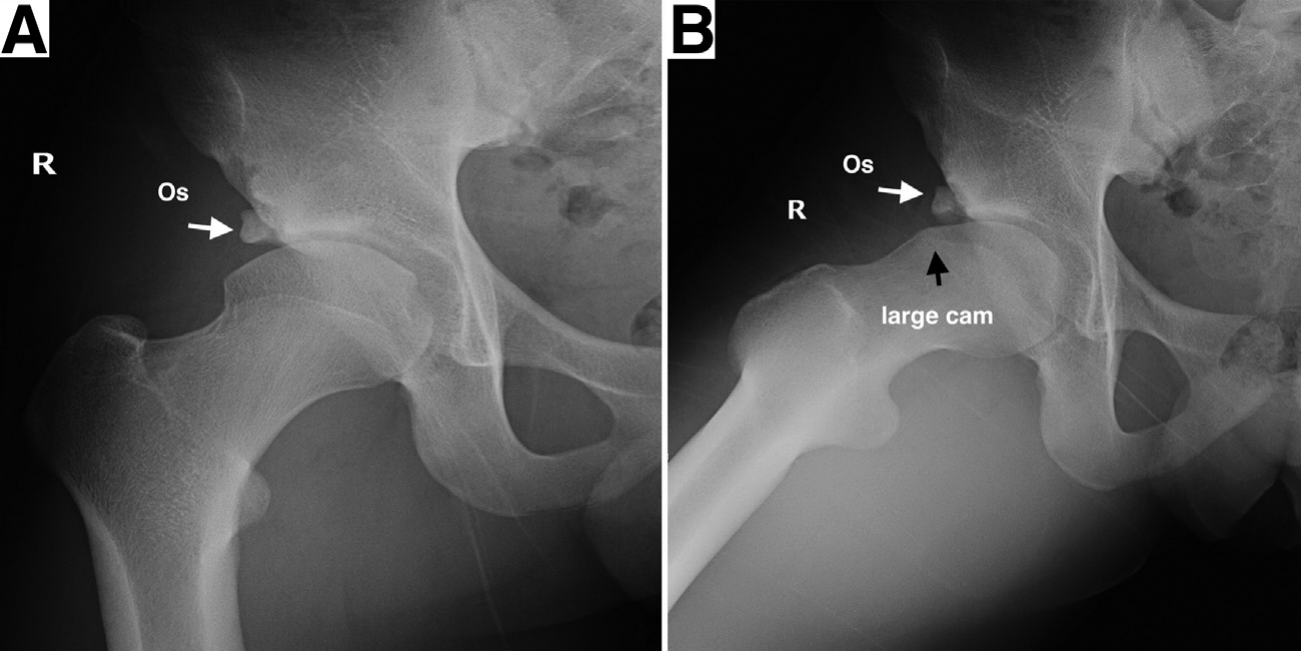
Anteroposterior view (A) and Dunn lateral view (B) of the right hip representing os acetabuli (white arrow) in the setting of a large cam lesion (black arrow). (Os, os acetabuli; R, right)

**Fig 2:**
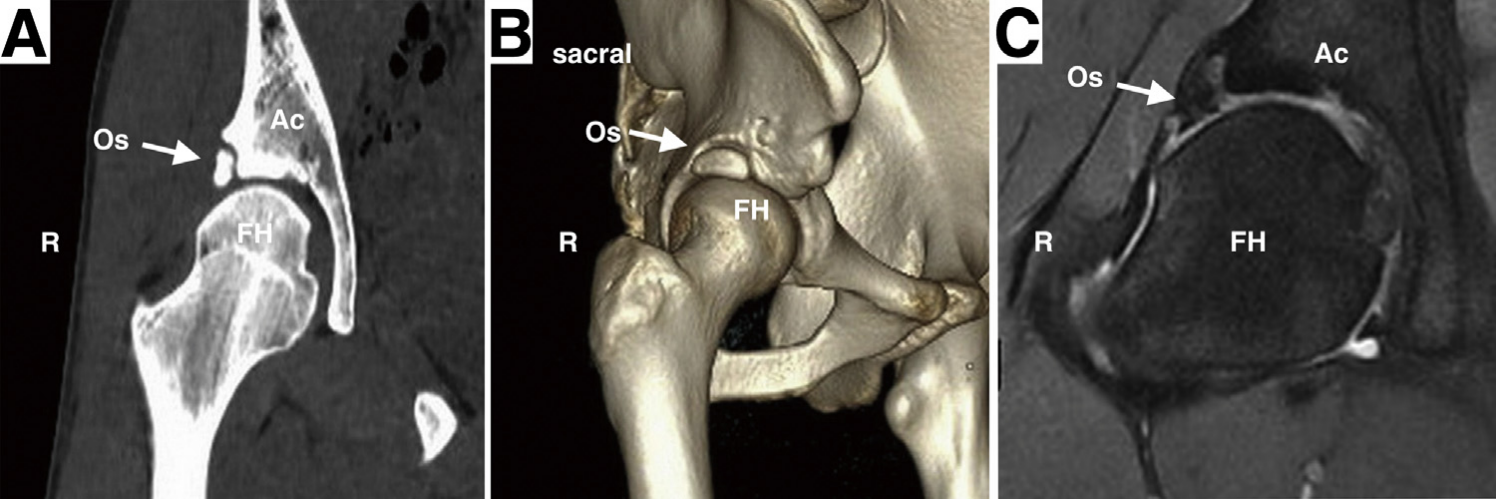
Preoperative CT and MRI of the right hip displaying os acetabuli (white arrow) and related injury. (A) Two-dimensional reconstructed coronal CT image showing the a bone fragment isolated from the acetabular rim. (B) Three-dimensional reconstructed CT image showing the spatial location of the Os acetabuli. (C) MRI image showing os acetabuli and related chondrolabral injure. (Ac, acetabulum; CT, computed tomography; FH, femoral head; MRI, magnetic resonance imaging; Os, os acetabuli.)

**Fig 3:**
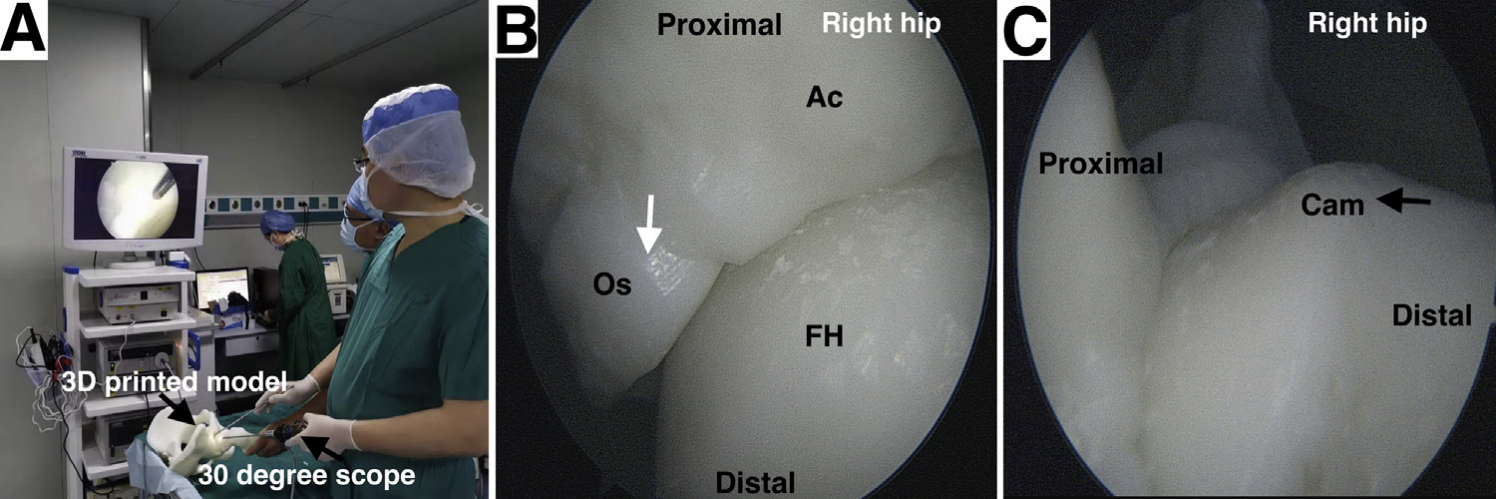
Preoperative arthroscopic simulation with a 30° scope showing the preview of os acetabuli and cam lesion on a 3D-printed model. (A) Overall setup of the preoperative arthroscopic simulation with a 3D-printed model. (B) Arthroscopic preview of acetabular rim and os acetabuli (white arrow). (C) Arthroscopic preview of cam lesion (black arrow). (3D, 3-dimensional; Ac, acetabulum; FH, femoral head; Os, os acetabuli.)

The preoperative evaluation should include an overview of the patient’s general health, medical history, and sports habits, as well as injury history and previous treatment experience. A physical examination, including anterior impingement provocative test and assessment of the range of motion, is necessary. A comprehensive radiographic evaluation is important in determining the surgical protocol. The anteroposterior pelvic radiographs can show the general condition of the joint and the relationship of the bone fragment to the lateral acetabular coverage. A Dunn view of the affected hip could facilitate the evaluation of cam deformities (Fig 1). A 3-dimensional computed tomography image can provide better visualization of the spatial location and size of the bone fragments, as well as the morphology of the subspinal region and cam lesions. A magnetic resonance imaging scan provides the information of injury of labrum and cartilage (Fig 2). A3-dimensional printed pelvic model based on the patient’s preoperative computed tomography data can be obtained to further visualize the hip deformity, on which a preoperative simulation of the arthroscopic procedure could be performed (Fig 3).

### Operative Technique

The patient is placed supine on the fracture table with the operated limb in a neutral abduction–adduction position with maximal internal rotation and 5° to 10° hip flexion. A 30° arthroscope is placed through the anterolateral portal to reach the extracapsular space of the hip, and then instruments are placed through the mid-anterior portal to expose the iliofemoral ligament and the anterior capsule. A longitudinal capsulotomy could be performed between the medial and lateral bundles of the iliofemoral ligament to expose the cam lesion in the peripheral compartment of the hip joint. Gentle traction could be applied after capsulotomy completed until the joint space reaches 8 to 10 mm. Arthroscopic exploration of the central compartment could reveal the severity of chondrolabral injury.

**Fig 4:**
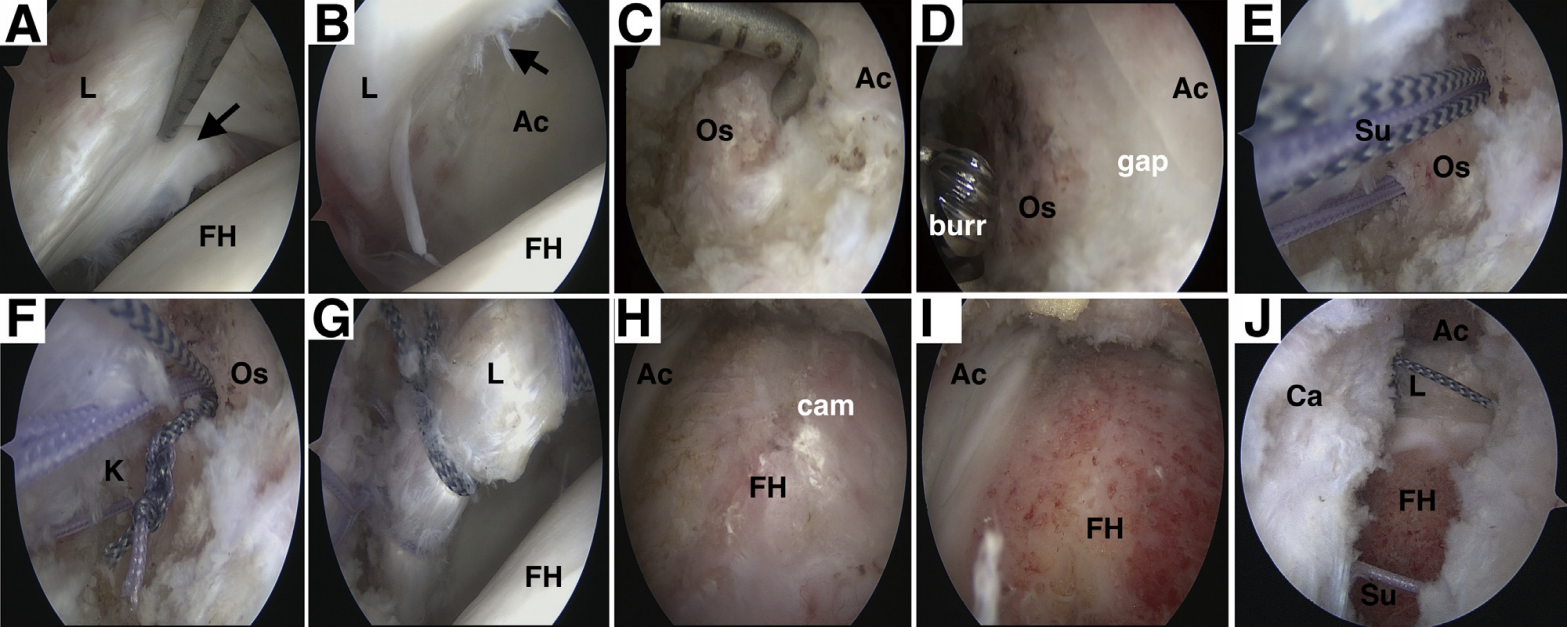
Arthroscopic views of the right hip from the mid-anterior portal with a 30° scope showing the main procedures and intraoperative findings during arthroscopic management of os acetabuli. (A) Arthroscopic view showing torn labrum is probed. (B) Arthroscopic view from the central compartment showing the chondrolabral damage related to os acetabuli. (C) Arthroscopic view showing probe hook and soft tissue gap between os acetabuli and acetabulum. (D) Arthroscopic view showing the trimming of bone fragment and acetabular rim with a dynamic burr. (E) Arthroscopic view showing 2 suture anchors implanted penetrating the bone fragment. (F) Arthroscopic view showing sutures knotted (K) in double pulley fashion to fix the os acetabuli. (G) Arthroscopic view showing the reattachment of the torn labrum with another suture of the same anchor. (H) Arthroscopic view showing the large cam lesion was exposed. (I) Arthroscopic view showing the smooth surface of head-neck junction after resection of cam lesion. (J) Arthroscopic view showing capsular closure with #2 non-absorbable sutures in a side-to-side fashion. (Ac, acetabulum; Ca, capsule; FH, femoral head; K, knot; L, labrum; Os, os acetabuli; Su, sutures.)

**Fig 5:**
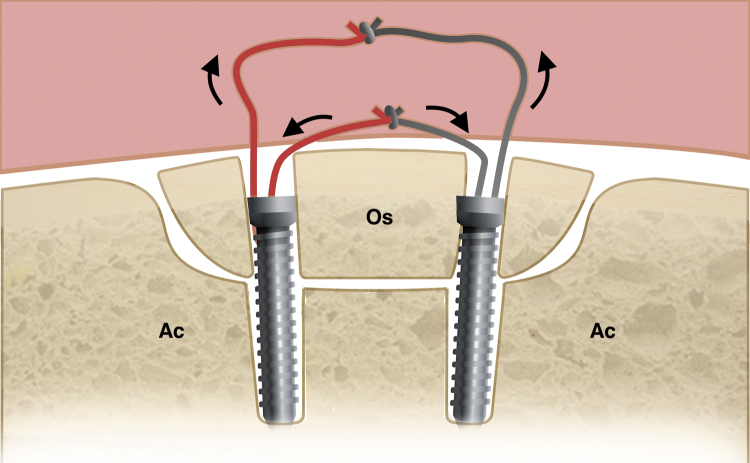
The schematic diagram showing the anchor implant and suture knotting in the suture fixation of os acetabuli with double-pulley technique. (Ac, acetabulum; Os, os acetabuli.)

Subsequent fixation of os acetabuli could be performed according to the surgical plan. First, the rim of os acetabuli and hypertrophic subspine is exposed and trimmed with a 5.5-mm dynamic burr (Smith & Nephew, Andover, MA). Second, 2 absorbable 3.0-mm Gryphon anchors (DePuy Mitek, Raynham, MA) are placed penetrating the bone fragments and anchoring to the acetabular bone bed. One limb of one suture from each anchor is knotted at the end and passed down onto the rim of os acetabuli, and then the free limbs of the suture are tied down with a standard sliding knot to compress the bone fragments (double-pulley technique). Another suture of anchors is used for the cerclage of the labrum. Additional anchors could be used to repair the torn labrum in traditional fashion if necessary. With traction force released, the cam lesion could be comprehensively evaluated with the hip flexed to 0° to 60°. The cam resection could be performed with a 5.5-mm dynamic burr (Smith & Nephew). Intraoperative dynamic impingement check and fluoroscopy are used to confirm the elimination of hip impingement. Finally, the joint capsule is closed with #2 nonabsorbable sutures in a side-to-side fashion. Figure 4 demonstrates the main procedures of arthroscopy, and the schematic diagram of the double pulley technique is shown in Figure 5. Table 1 details the pearls and pitfalls of our technique.

**Table 1:** Technical Pearls and Pitfalls

Pearls	Pitfalls
Determine the location and extent of the bone fragment Expose and assess the stability of bone fragment Partially resect the bone fragment with a dynamic burr to create a flat bone surface Make a deeper predrill hole than routine facilities anchoring into the acetabular bone bed Use tap-in anchors with small diameter Use double-loaded suture anchor Retrieve suture limbs in one portal Preserve the labrum	Insufficient exploration of bone fragment Too shallow of a predrill hole cannot penetrate bone fragment Avoid using anchors with a large diameter Prevent tangling sutures Insufficient correction of cam deformity Make sure the proper angle of anchor placing

### Postoperative Rehabilitation and Return to Play

**Fig 6:**
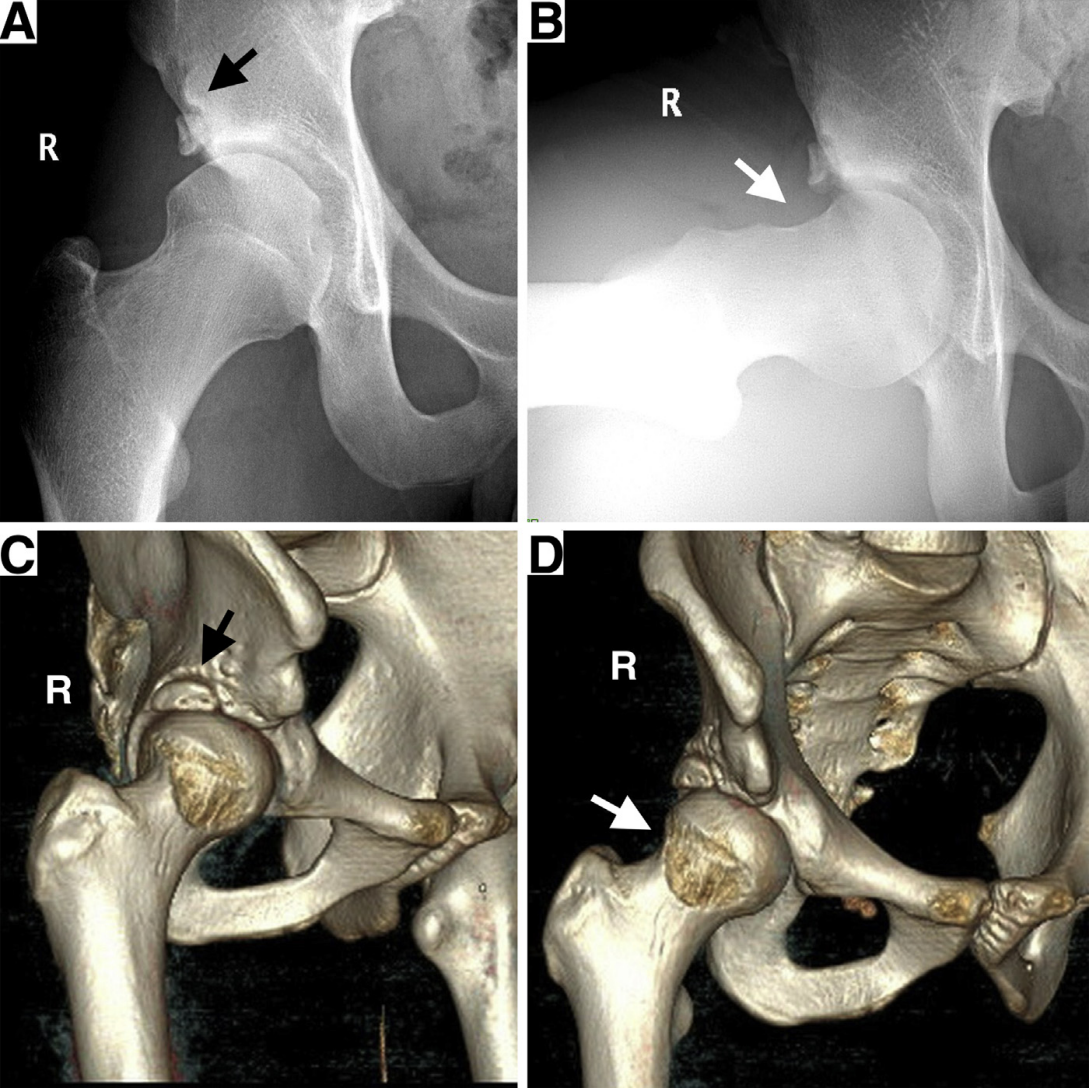
The postoperative anteroposterior view (A) and Dunn view (B) of the right hip plain radiography showing the os acetabuli was well secured (black arrow) and sufficient correction of cam lesion was made (white arrow). The postoperative 3-dimensional computed tomography image with different rotating views (C and D) showing proper fixation of os acetabuli (black arrow) and the complete correction of cam lesion (white arrow).

A relatively conservative postoperative rehabilitation is advised, with 90° of hip flexion at 4 weeks, 120° of hip flexion at 6 weeks, and weight-bearing restriction with crutches for 6 weeks. Half-squat could be resumed in 3 months postoperatively. Competing sports activity is forbidden until 6 months after surgery. Radiographic follow-up is indicated 3 months postoperatively to ensure proper fixation of the bone fragment and decompression of hip impingement (Fig 6).

## Discussion

Although the origin of the os acetabuli is controversial, the presence of bone fragment could be related to the femoroacetabular impingement, and the micromotion of bone fragment and the following recurrent microinjuries could result in the pain and dysfunction of the hip joint. Randelli et al.^8^ and Singh and O’Donnell^9^ independently reported a prevalence of approximately 7% in the young active male athletic population, which indicates os acetabuli is an issue that should not be neglected. Based on the recognition of the etiology and pathogenesis, the treatment for os acetabuli lies in 2 aspects, first, addressing impingement between the femoral head and acetabular rim; second, eliminating the micromotion of the bone fragment. Resection of cam lesion is definitively important, which could decrease the collision from the femoral head–neck junction and provide a good biomechanical environment for the resecure of os acetabuli. The removal or fixation of the os acetabuli mainly depends on the acetabular coverage of the hip joint. In cases that the bone fragment is not involved in the acetabular coverage, a good outcome could be expected with resection of the bone fragment.^5^ Larson and Stone^6^ noted the importance of measuring the LCEA when determining the management of os acetabuli. When the LCEA is >25° and the anterior center edge angle is >20° without fragments, the fragments can be completely resected. If the removal of fragments results in an LCEA <25° and an anterior center edge angle <20°, the fragment should be retained or partially resected.

The technique of using metal screws for the fixation of the bone fragment has been reported by several authors.^10^ Cuéllar et al.^11^ reported a technique of fixation of os acetabuli using hollow screws. Pérez et al.^12^ and DeFroda et al.^13^ reported the suture-on-screw technique for os acetabuli fixation and labral repair simultaneously. Essilfie et al.^14^ reported a hybrid technique to fix acetabular rim fractures and labrums with metal screws and suture anchors, respectively. This technique theoretically provides rigid fixation for os acetabuli, but it also has some drawbacks, first, it could be a technical challenge to perform an arthroscopic fixation using common instruments of routine traumatic orthopaedics. Second, the bone fragment could split into pieces if the screw has a relatively large diameter. Third, potential breakage or loosening of the metal screw, and secondary damage to the hip joint. Therefore, we adapted and modified the arthroscopic double-pulley technique for the bony Bankart lesion of the shoulder joint, and apply it in the arthroscopic fixation of os acetabuli in the hip joint. Different from the conventional rigid screw fixation, we call it suture fixation.

In this technique, 2 absorbable anchors were used to penetrate the fragment anchoring into the acetabular bone bed to provide primary stability, followed by sutures knotted in a double-pulley fashion to further secure the fragment. This technique has several advantages. First, it avoids any metal being implanted and eliminates its relative complications. Second, using absorbable suture anchors with smaller diameters could decrease the risk of breakdown of the bone fragments. Third, this technique does not separate and clean the gap between bone fragment and bone bed, with anchor penetrating the fragment directly and suture knotted in a double- pulley fashion, which avoids the difficulty of circumscribing the bone with sutures, and also provide compress stress to bone fragment. In addition, as each anchor loaded double-stranded sutures, one suture is used for fixation of bone fragment, and another suture could be used to reattach the torn labrum. This is also the difference between our technology and that proposed by Lund.^15^ Advantages and limitations of our technique are detailed in Table 2.

**Table 2:** Advantages and Disadvantages of the Technique

Advantages	Disadvantages
Avoid any metal implanted and eliminate the relative complication. Using absorbable suture anchors with smaller diameters decrease the risk of breakdown of the bone fragments. Avoid the difficulty of circumscribing the bone with sutures. Double-loaded suture anchor could be used to reattach the labrum simultaneously	Not rigid fixation The risk of breakage of absorbable anchor Difficult arthroscopic technique

As shown in our short-term follow-up, patients with painful os acetabuli who underwent hip arthroscopic surgery with this technique could improve their hip function and resumed high-competing sports. In summary, using absorbable anchors to penetrate the bone fragment and secure it in a double-pulley technique provides a feasible and effective arthroscopic suture fixation method for os acetabuli.

## Supplementary Data

ICMJE author disclosure forms

Video 1 Arthroscopic suture fixation of os acetabuli with absorbable suture anchors—a double-pulley technique.
